# The Role of Alveolar Epithelial Cells in Initiating and Shaping Pulmonary Immune Responses: Communication between Innate and Adaptive Immune Systems

**DOI:** 10.1371/journal.pone.0032125

**Published:** 2012-02-29

**Authors:** Olga D. Chuquimia, Dagbjort H. Petursdottir, Muhammad J. Rahman, Katharina Hartl, Mahavir Singh, Carmen Fernández

**Affiliations:** 1 Department of Immunology, Wenner-Gren Institute, Stockholm University, Stockholm, Sweden; 2 Lionex Diagnostics and Therapeutics GmbH, Braunschweig, Germany; Albany Medical College, United States of America

## Abstract

Macrophages and dendritic cells have been recognized as key players in the defense against mycobacterial infection. However, more recently, other cells in the lungs such as alveolar epithelial cells (AEC) have been found to play important roles in the defense and pathogenesis of infection. In the present study we first compared AEC with pulmonary macrophages (PuM) isolated from mice in their ability to internalize and control *Bacillus Calmette-Guérin* (BCG) growth and their capacity as APCs. AEC were able to internalize and control bacterial growth as well as present antigen to primed T cells. Secondly, we compared both cell types in their capacity to secrete cytokines and chemokines upon stimulation with various molecules including mycobacterial products. Activated PuM and AEC displayed different patterns of secretion. Finally, we analyzed the profile of response of AEC to diverse stimuli. AEC responded to both microbial and internal stimuli exemplified by TLR ligands and IFNs, respectively. The response included synthesis by AEC of several factors, known to have various effects in other cells. Interestingly, TNF could stimulate the production of CCL2/MCP-1. Since MCP-1 plays a role in the recruitment of monocytes and macrophages to sites of infection and macrophages are the main producers of TNF, we speculate that both cell types can stimulate each other. Also, another cell-cell interaction was suggested when IFNs (produced mainly by lymphocytes) were able to induce expression of chemokines (IP-10 and RANTES) by AEC involved in the recruitment of circulating lymphocytes to areas of injury, inflammation, or viral infection. In the current paper we confirm previous data on the capacity of AEC regarding internalization of mycobacteria and their role as APC, and extend the knowledge of AEC as a multifunctional cell type by assessing the secretion of a broad array of factors in response to several different types of stimuli.

## Introduction

Tuberculosis (TB) is still one of the most devastating diseases affecting both humans and animals [1], [2]. Transmission often takes place via aerosol from individuals with the active form of pulmonary TB. This, together with the fact that *Mycobacterium tuberculosis* (Mtb) has a marked tropism for the lungs, makes pulmonary TB the most frequent form of the disease and the lungs the target organ [3]. Thus, interactions between mycobacteria and different host target cells in the respiratory mucosa, dictate the outcome of mycobacterial infection in man ranging from an asymptomatic infection to a life-threatening disease. In these interactions both innate and adaptive immune responses play critical roles.

Disease can be prevented in two ways; a) the innate immune system alone can be able to impede bacterial invasion and infection, b) if infection takes place, two alternatives can occur, either the host adaptive immune system is able to control bacterial replication or it will fail in this process. The host will then develop active disease and recover or eventually succumb. Moreover, upon infection, Mtb is able to reprogram its gene expression, preventing the immune system from totally eliminating the microorganism leading to latent infection of the host [4], [5]. The identification of the mechanisms controlling Mtb adaptation to the intracellular environment remains to be solved.

Macrophages and dendritic cells (DC) have long been recognized as key players in the defense against mycobacterial infection but also important in the pathogenesis of TB and the physiology of latent Mtb infection [6]. However, more recently, other cell types, such as adipocytes, fibroblasts, endothelial cells and epithelial cells have also been found to play important roles in the defense and pathogenesis of infection and even been identified as cellular niches for latent Mtb [7]. Moreover, protection against respiratory infection is also provided by the physical barrier formed by alveolar epithelial cells (AEC). AEC are abundant in number and line the pulmonary airways and alveoli. There are two types namely, AEC I and AEC II. AEC I are the epithelial component of the thin air-blood barrier and comprise approximately 95% of the alveolar surface area [8], [9]. The AEC II cover approximately 4% of the mammalian alveolar surface but constitute 15% of all lung cells [8]–[11]. AEC II, and to a lesser extent AEC I, have been shown to be important effector cells in inflammatory responses. Furthermore, AEC II perform a variety of important functions within the lung, including regulation of surfactant metabolism, ion transport, and alveolar repair in response to injury [12], [13]. Due to the location of these epithelial cells during the initial steps of infection, the chance that a pathogen encounters AEC II is much greater than encountering a macrophage. Upon infection, AEC II can release a number of antimicrobial molecules, cytokines and chemokines [14]. This network of mediators may contribute to migration of monocytes and macrophages to the site of infection and also promote activation of their antimicrobial activity. Moreover, murine and human AEC II express MHC class II molecules on their surface and have been proposed to be able to present antigens to CD4 T cells [15]–[17].

Compared to macrophages much less is known about Mtb and epithelial cell interactions. Several factors contribute to this. AEC II comprise only 15% of all lung cells which makes it difficult to attribute specific functions to type II cells from studies of whole lungs or mixed cell cultures [18]. Purification of these cells from lung tissue is not a trivial procedure. In humans, lung tissue is obtained from biopsies of patients affected with various diseases. Samples are then relatively big but even if injured material is disregarded, it is not absolutely certain that the rest of the considered “healthy” organ is not affected. In mice, healthy tissue can be obtained but the sample size is small and the procedure long and costly. Unfortunately, there is not a cell line that exhibits the full range of known type II cell functions. Results are often contradictory.

In the present study we have first compared AEC with PuM in their ability to internalize and control Bacillus Calmette-Guérin (BCG) growth and their capacity to present antigen to antigen-primed T cells. Secondly, we have compared both cell types in their capacity to secrete a number of cytokines and chemokines upon stimulation with various molecules including mycobacterial products. Finally, we have analyzed the profile of response of AEC to diverse stimuli in an attempt to understand the role of these cells in the defense of the respiratory tract.

## Results

### Purification of alveolar epithelial cells (AEC) and pulmonary macrophages (PuM)

**Figure 1:**
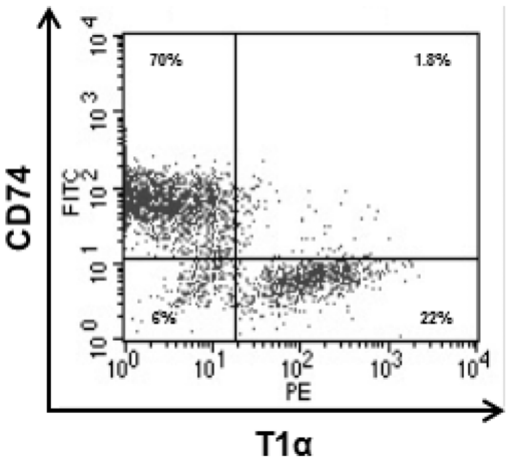
Flow cytometry analysis of freshly isolated AEC from mouse lungs. Total lung cells were obtained by using Corti's protocol with some modifications as described in Material and Methods. Leukocytes were first depleted with anti-CD45 microbeads and subsequently, the CD45**^−^** cells were depleted of contaminating endothelial cells using anti-CD146 microbeads. The remaining CD45^−^CD146^−^ cells were considered to be AEC. These cells were fixed and stained intracellularly with antibodies to CD74 (AEC II marker) and podoplanin (T1α) (AEC I marker). A representative dot plot of flow cytometry analysis of CD74 and T1α expression in freshly isolated AEC from three independent experiments is shown. Percentage numbers represent gated cells from total cells.

AEC were purified as described in Materials and Methods by depletion of CD45^+^ and CD146^+^ cells. The phenotypic characterization of freshly isolated alveolar epithelial cells (AEC) was determined by flow cytometry based on intracellular staining of CD74 a marker for AEC II [19] and podoplanin (T1α) a marker for AEC I [20]. In average, 92–95% of the cells isolated displayed the AEC phenotype where approximately 70% were AEC II and 22% were AEC I (Figure 1). A minor fraction, was found to be positive for CD45 (2%) and CD31 (3%). Cells purified in this manner will be named AEC in this study. The phenotype of PuM was determined by staining with F4/80 and the purity was of 98%.

### Mycobacterial uptake and intracellular growth. Comparison of AEC and PuM

**Figure 2:**
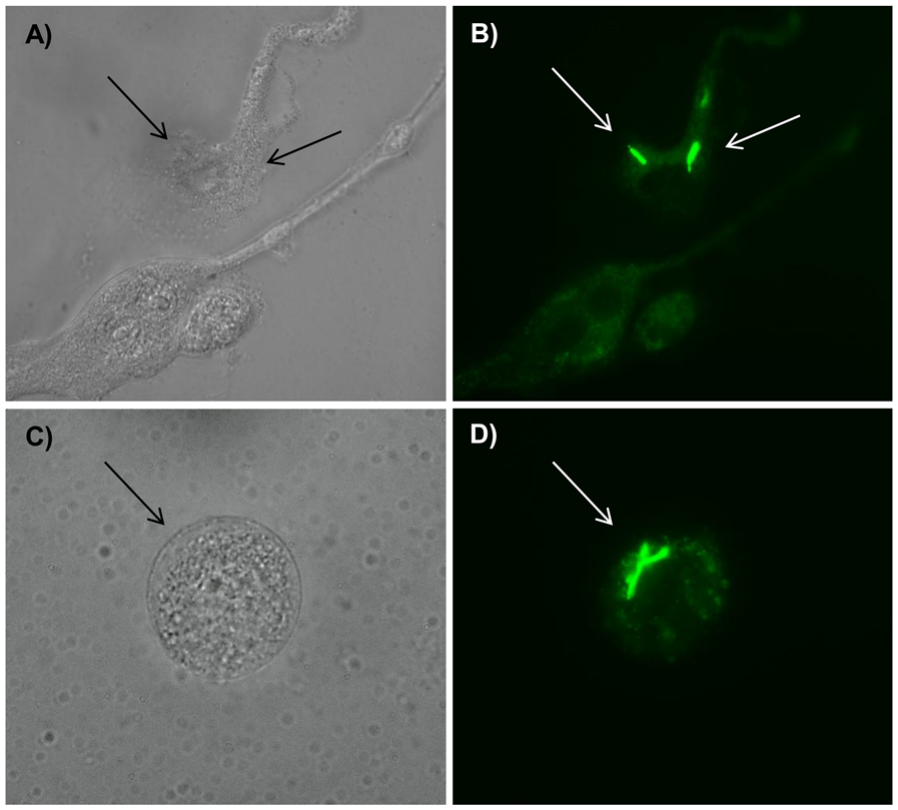
Uptake of mycobacteria by AEC and PuM. Isolated AEC (A and B) and PuM (C and D) were cultured on cover glass at a concentration of 1×10^5^ cells per well as described in Material and Methods. After overnight culture in medium without antibiotics, the cells were infected with GFP-BCG at a MOI of 1∶100 (cell∶bacteria) for 4 h at 37°C in RPMI medium without antibiotics. To kill all extracellular remained bacteria, the cells were treated with gentamicin for 1 h, washed 3 times and finally incubated for 72 h in RPMI without antibiotics. After that, the cover glasses with infected cells were fixed, mounted and observed under white light (A and C) and green light (B and D) in a fluorescence microscope. Magnification 1000×.

PuM have been suggested to be most important in the control of pulmonary bacterial infection. To understand the role of AEC in this issue, we evaluated the capacity of both cell types in the uptake and control of intracellular growth of mycobacteria. Primary AEC and PuM were infected with GFP-BCG, a recombinant BCG expressing green fluorescent protein (GFP) and luxAB [21], at a multiplicity of infection (MOI) of 10∶1 or 100∶1 (bacteria∶cell) for 4 h. Upon removal of extracellular bacteria, uptake (0 h) and intracellular growth (72 h) were determined by measuring the amounts of relative luminescence units (RLU) in cell lysates. Even if PuM displayed a higher capacity than AEC, our results demonstrated that AEC were also able to internalize mycobacteria as assessed by microscopy (Figure 2) and RLU determinations Table 1 (AEC: 195±37 versus PuM: 676±94). The increase in RLU between 0 and 72 h was also measured to determine intracellular bacterial growth. After 72 h, the increase in RLU in cell free medium was 6 fold, while in AEC and PuM lysates the increase was 4.6 and 2.7 fold, respectively (Table 1). This demonstrated that both cell types were able to control intracellular mycobacterial growth but that also in this aspect PuM were more efficient than AEC.

**Table 1:** Mycobacterial uptake and intracellular growth by AEC and PuM.

	AEC		PuM
**0 h**	195±37	**(3.5×)**	676±94
	**(4.6×)**		**(2.7×)**
**72 h**	908±142		1885±254

Data are expressed in relative luminescence units (RLU). Uptake is measured at 0 h and intracellular growth at 72 h. Increase in RLU from GFP-BCG cultured in cell free medium was at 72 h, 6 fold of the measured at 0 h. This value was used as a reference to bacterial growth. Data are an average from four different experiments and expressed as mean ± SEM, n = 15.

### Antigen presentation by AEC

**Figure 3:**
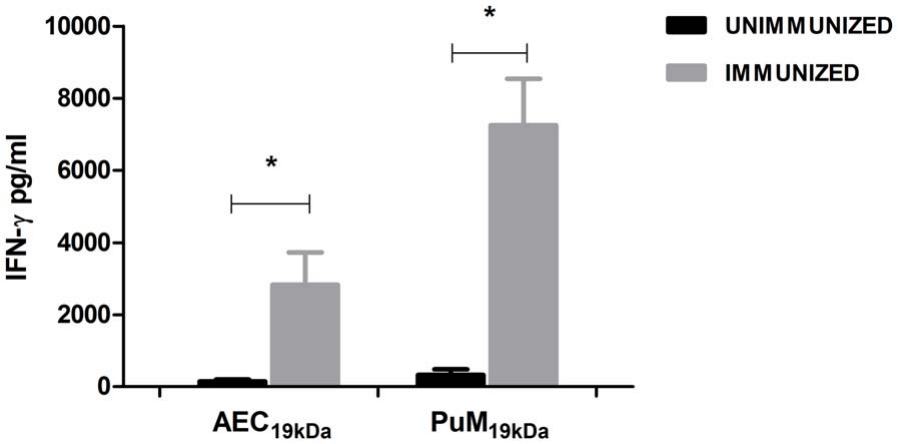
Antigen presentation by AEC. IFN-γ levels after *in vitro* restimulation of splenocytes from Ag19 kDa immunized mice. As APC we used AEC (AEC_19 kDa_) and PuM (PuM_19 kDa_) pulsed with Ag19 kDa as described in Materials and Methods. One week after the last immunization with Ag19 kDa, mice were sacrificed and splenocytes from immunized and unimmunized mice were co-cultured with AEC_19 kDa_ and PuM_19 kDa_ for 72 h. Data are expressed as mean ± SD from 3 mice per group. A representative of two independent experiments is shown. * represents levels significantly different from unimmunized control, p<0.05.

Since we found that purified AEC were able to internalize mycobacteria, we next examined their capacity to present antigen. This is important because it has been described that AEC II can express MHC class II antigens but possibly lack other co-stimulatory molecules [16], [17], [22] and therefore, it is questionable if AEC II can participate in adaptive immune responses as non-classical APCs. Even if in our AEC population only 70% of the cells were AEC II, we addressed this question by testing the ability of AEC and PuM pulsed for 24 h with the mycobacterial antigen 19 kDa (AEC_19 kDa_ and PuM_19 kDa_, respectively) to present antigen to T cells primed with the same antigen. To avoid interferences, free antigen was eliminated by thorough washing. Splenocytes from non-immunized mice and mice immunized with the same antigen were co-cultured with AEC_19 kDa_ and PuM_19 kDa_ to assess the functionality of APC on the primed T cells. As a read out, IFN-γ was measured in the culture supernatants. Pulsed AEC were clearly able to stimulate splenocytes from 19 kDa immunized mice even if to a lower degree than PuM demonstrating that AEC could collaborate with T cells in the adaptive response to mycobacterial infection (Figure 3).

### Comparison of cytokine and chemokine production by AEC and PuM

To investigate the possible participation of AEC and PuM in the local immune response either directly or indirectly by influencing other cells in their near vicinity, we used various stimuli. We chose the cytokines TNF and IFN-γ, because both are important in the control of mycobacterial infection; LPS because it is an inducer of innate responses and a ligand for TLR 4 present on both cell types [23], [24] and the BCG products: Lys-BCG (lysate) and (heat killed) HK-BCG. We tested for the production of the cytokines IL-12 and TNF and the chemokines MIP-2 and MCP-1 described to be secreted by macrophages and AEC cells [25], [26]. We performed our experiments in transformed cell lines and in primary cells isolated from healthy lung tissue.

Our results (Table 2) show that AEC were the main producers of MCP-1 while PuM were the main producers of MIP-2. TNF was a good inducer of MCP-1 by AEC. Since macrophages are the main producers of TNF, these results indicate a possible influence of PuM on AEC. HK-BCG was a better stimulant that the lysate suggesting that the major stimulating ligands may be still present in the BCG cell wall or because HK-BCG was given in a particulate form. Production of TNF and IL-12 was only observed in PuM upon stimulation with LPS (data not shown).

**Table 2:** Chemokine production by primary AEC and PuM after *in vitro* stimulation.

	MCP-1		MIP-2	
Stimulus	AEC	PuM	AEC	PuM
-	1750±510	300±40	ND	ND
HK-BCG	**7100±730**	700±90	140±6	210±240
Lys-BCG	2700±270	500±40	90±6	600±30
TNF	**14200±1100**	220±10	60	300±70
IFN-γ	3000±540	580±60	ND	ND
LPS	**14800±3400**	1400±30	600±90	**21500±5500**

Data are expressed in pg/ml of cytokine produced. A representative experiment from five independent experiments is shown. Values are given as mean ± SD n = 3–4. ND: not detected.

The results using cell lines corroborated the results obtained with the primary cells but in general, primary cells were better responders to the various stimuli (Table 3).

**Table 3:** Chemokine production by the cell lines T7 and MH-S after *in vitro* stimulation.

	MCP-1		MIP-2	
Stimulus	T7	MH-S	T7	MH-S
-	11000±2900	11000±3000	ND	700±30
HK-BCG	10800±1500	18000±2800	ND	**3500±2300**
Lys-BCG	11500±800	12000±§300	ND	700±20
TNF	**32000±3200**	9800±4100	ND	1000±100
IFN-γ	11000±1900	17000±3200	ND	240±40
LPS	**19000±1200**	19000±3300	ND	**10300±830**

Data are expressed in pg/ml of cytokine produced. A representative experiment from five independent experiments is shown. Values are given as mean ± SD n = 3–4. ND: not detected.

### Induction of MMP-9

We also tested for the production of MMP-9 (Table 4) because this molecule has recently been described as important in granuloma formation and in the control of mycobacterial infections [27]–[29]. In contrast to studies done by other groups using macrophages of a different origin [29], PuM were not good secretors of MMP-9. However, AEC were good secretors of MMP-9 upon stimulation with TNF and LPS and to a lesser extent with HK-BCG. At present we cannot provide a valuable explanation for the low responsiveness of the PuM other than the lung environment may have made them unresponsive to the stimuli used in our study. The cell lines were not good responders (data not shown).

**Table 4:** MMP-9 production by primary cells.

Stimulus	-	HK-BCG	Lys-BCG	TNF	IFN-γ	LPS
AEC	2600±350	4100±1700	3000±650	**15100**±900	500±70	**9700±300**
PuM	ND	ND	ND	ND	ND	ND

Data are expressed in pg/ml of cytokine produced by primary cells. A representative experiment from five independent experiments is shown. Values are given as mean ± SD n = 3–4. ND: not detected.

### Secretion of cytokines, chemokines and growth factors by stimulated AEC

After the comparison between AEC and PuM in secretion of the ILs and chemokines named above, we were interested in determining a more complete profile of molecules secreted by AEC. We performed further analyses of AEC derived supernatants using the R&D Mouse Proteome Profiler array. We tested medium from unstimulated cultures and from AEC stimulated with LPS, Pam_3_Cys-Ser-(Lys)_4_ trihydrochloride (Pam3), Flagellin, TNF, IFN-α, HK-BCG, Lys-BCG, and the Mtb products HK-Mtb (from the virulent strain H37Rv) and HK-SO2 (from the Mtb attenuated phoP mutant strain SO2).

**Figure 4:**
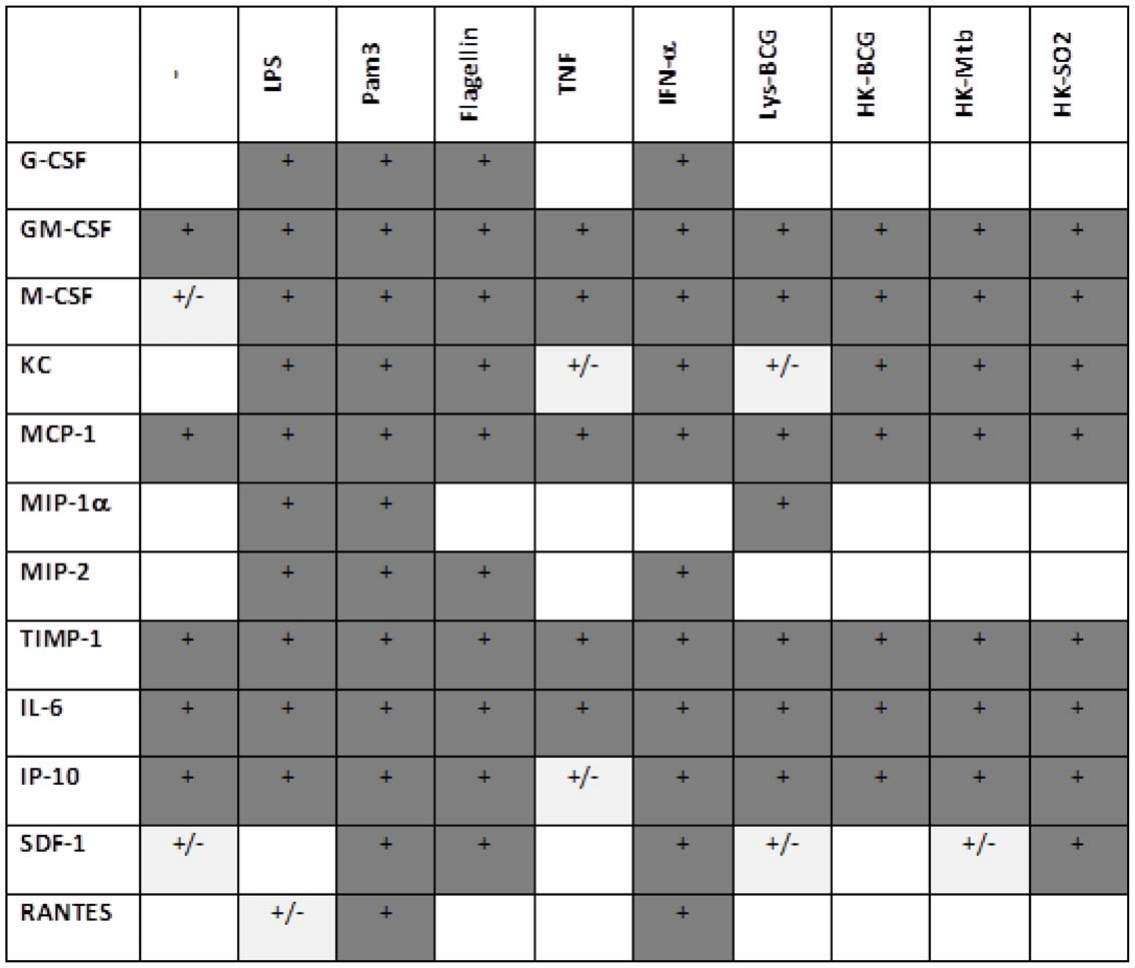
Cytokine/chemokine profiling on supernatants from AEC. AEC (5×10^4^ cells per well) were stimulated with the indicated stimuli for 24 h. Supernatants were incubated on membranes from a mouse Cytokine Array Panel A Proteome Profiler kit (R&D systems) according to the manufacturer's instructions. The figure shows a+representing a spot with similar or greater density than control spots, whereas +/− indicates a spot with lower density than control spots.

From the 40 cytokines, chemokines and growth factors included in the array, we observed the presence of 12 of them, namely G-CSF, GM-CSF, M-CSF, KC, MCP-1, CCL3/MIP-1α, MIP-2, tissue inhibitor of metalloproteinases (TIMP)-1, IL-6, IP-10, CXCL12/stromal cell-derived factor (SDF)-1 and RANTES. Some of the factors were produced constitutively (GM-CSF, MCP-1, TIMP-1, IL-6 and IP-10) since they were observed in supernatants from unstimulated cultures, while others (G-CSF, M-CSF, KC, MIP-1α MIP-2, SDF-1 and RANTES) were only detected upon stimulation. The various stimuli induced a distinct profile suggesting that AEC could activate different pathways depending on the stimuli used (Figure 4).

The Mtb products, HK-Mtb and HK-SO2 were included in this study to explore possible differences between Mtb and BCG in their stimulatory capacity. We found their profile to be very similar with the exception of SDF-1 induction. However, after measuring SDF-1 by quantitative ELISA, no difference was observed in the SDF-1 levels in supernatants from cells stimulated with BCG or Mtb products (not shown). Since ELISA is a quantitative method and the proteome profiler is not, we concluded that the results from the ELISA assay were more reliable and therefore HK-Mtb, HK-SO2 and HK-BCG exhibited similar stimulatory capacities.

**Figure 5:**
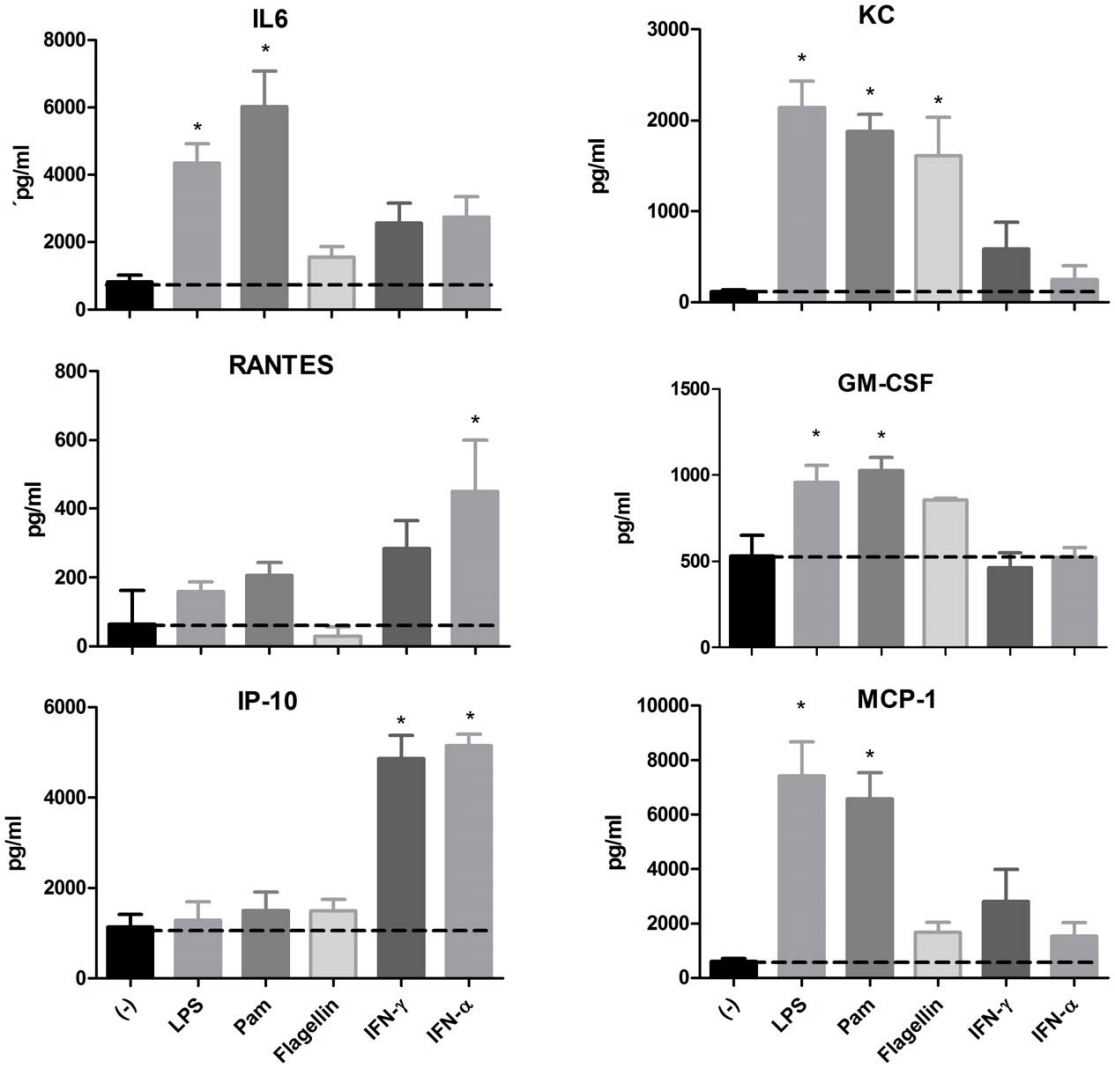
Quantitative measurements of selected factors in supernatants from AEC. AEC (5×10^4^ cells per well) were stimulated with the indicated stimuli for 24 h. IL-6, KC, GM-CSF, RANTES, MCP-1, and IP-10 levels were measured in cell culture supernatants using ELISA. Values are expressed as means ± SD, from 3 independent experiments. * represents levels significantly different from unstimulated control, p<0.05. The dotted line indicates the concentration in supernatants from unstimulated cells.

To determine if the levels of factors produced constitutively could be increased upon stimulation we selected some factors to determine their contents by using quantitative ELISA. The factors selected were: IL-6, KC, RANTES, GM-CSF, M-CSF, IP-10, and MCP-1. As stimuli we chose the three TLR ligands described above as well as IFN-α and IFN-γ to analyze different pathways of stimulation. It can be observed that the TLR ligands and IFNs displayed different profiles. The chemokines MCP-1 and KC, and the pleiotropic cytokine IL-6 showed the strongest induction after induction via TLRs, whereas the production of IP-10 and RANTES were predominantly induced by IFNs. Also of interest is to point out that the profile of the three TLR ligands tested were different with flagellin being the ligand with the lowest stimulatory capacity (Figure 5). This was also the case at other concentrations tested (data not shown).

The growth factor GM-CSF increased twofold after stimulation with LPS and Pam3, whereas M-CSF could not be induced by the stimuli used (not shown). Furthermore, M-CSF was almost undetectable by ELISA (15–30 pg/ml), indicating that GM-CSF is the dominant growth factor secreted by AEC under these conditions.

## Discussion

DC and macrophages have been considered to be the first mediators of inflammatory responses in the lungs as well as being active in connecting to the adaptive immune system as APC. However, other cell types such as AEC may also be extremely important since they are more abundant in the lungs than PuM and, due to their more external localization, are possibly earlier than PuM in the contact with external aggressions. In this study, we first compared the responses of AEC and PuM. What we called PuM are interstitial and intravascular macrophages. We favor the hypothesis that the first cells in contact with microbial aggression or other types of insult are the epithelial cells and in particular the AEC II. These cells are possibly acting as sentinels establishing the basis for a successful communication with other cell types in the defense against infection. Since it has also been shown that AEC are able to respond to different types of aggressions, we reasoned that AEC would be important in the first line of defence against mycobacterial infections. Therefore, in this study we aimed to provide a better understanding of AEC responses against various stimuli including mycobacterial products. We have tested different types of stimuli from various sources i.e. lymphocyte-derived (cytokines), microbial (TLR ligands) and mycobacterial (virulent and non-virulent) products to cover a broad spectrum of activation and we measured in vitro a number of cytokines and chemokines to get an idea of the potential of AEC in the activation and communication with other cells in the lung tissue.

Initially, we compared the activity of healthy primary AEC and PuM regarding uptake of BCG and control of intracellular growth. Even if PuM displayed in both cases a higher capacity than AEC, our results demonstrated that AEC were also able to internalize and control bacterial growth. We also provide *ex vivo* evidence that AEC had the capacity to take up, process and present antigen as demonstrated by the ability of *in vitro* pulsed AEC to present the mycobacterial antigen 19 kDa to splenocytes derived from 19 kDa primed animals. However, AEC and T cells are localized in separate compartments. Thus, it is obvious that to be able to establish a successful collaboration with T lymphocytes, AEC have to promote the migration of these cells from the peripheral blood and other compartments to the lung tissue. Moreover, even if we and others have demonstrated the capacity of AEC as antigen presenting cells [17], [16] this capacity is probably secondary to other functions inherent to the AEC properties. In line with this, others have shown that primary AEC II express very low levels of the classical B7 co-stimulatory molecules [22], [30].

Collaboration of immune cell types is important to achieve effective protection. This may be particularly important in the protection of mucosal surfaces which are directly exposed to various insults such as toxins, allergens or microorganisms. In the present work, we pay special attention to the respiratory tract and the immune responses in the lungs against mycobacteria. Important in this issue is to promote the migration of mononuclear phagocytes from the peripheral blood to the lung tissue and airspace both to maintain the pool of lung phagocytes as well as to respond to lung insults. Although the process of inflammatory cell recruitment is complex and poorly understood, it is clear that the local generation of specific immune cell chemokines is required. To further accomplish an optimal immune response, communication between the innate and the adaptive branches of the immune system would be required. By comparing the capacity of AEC and PuM on the production of various factors we show a clearly different pattern of chemokines and cytokines secreted by activated PuM and AEC. PuM were found to produce lower levels of MCP-1 and higher levels of MIP-2 and KC (not shown) compared with stimulated AEC. Human AEC and human epithelial cells such as Clara cells have been described to produce the chemokine IL-8 [31], [32] functionally homologous to MIP-2 [26], [33], [34] and KC in mice [35]. Even if in this study the AEC were lower producers of MIP-2 and KC than the PuM, the amounts secreted were important and similar to that described for the secretion of IL-8 by human AEC [36]. Other groups have studied the production of MIP-2 by macrophages [25] but no systematic comparison between the two cell groups has been made before. Of course, this may also indicate differences in stimulation patterns and consequently not all aspects of innate responses can be translated bidirectional between humans and mice. Also, controversies between human and mouse studies may be related to the difficulties in obtaining healthy lung tissue from humans. It is obvious that, even if biopsies can be taken from physically separated locations, it cannot be guaranteed that presumptive bioactive molecules have never been in contact with the lung cells used in these studies. In contrast, even if work with mouse lung cells is more difficult due to other aspects, the health status of mouse lungs used for the preparation of AEC and PuM can be secured.

Another difficulty we faced in our work with mice is that unfortunately, there is not a cell line that exhibits the full range of known AEC functions. Results are often contradictory because the behavior of commercially available cell lines is different than the behavior of primary cells. We are certainly more confident with the results obtained from primary cells derived from healthy murine partners.

Of interest are the results showing the capacity of AEC to respond to both microbial and internal stimuli exemplified by TLR ligands and IFNs, respectively. The response included a broad array of cytokines, chemokines and growth and differentiation factors known to have various effects on other cells such as monocytes, macrophages, DC and T cells. Interesting is also that the AEC could be stimulated to a similar extent with mycobacterial products derived both from BCG and virulent and attenuated Mtb. Moreover, the finding that TNF could stimulate the production of MCP-1 by AEC to a similar extent as LPS is of particular interest because MCP-1 plays a role in the recruitment of monocytes and macrophages to sites of injury and infection and macrophages are the main producers of TNF. We also describe the production by AEC of the T lymphocyte chemokines IP-10 and RANTES. Thus, it is easy to speculate that AEC and other cell populations in the lungs can stimulate each other closing the circle of cell-cell interactions.

In summary, in the current paper we confirm previous data on the capacity of AEC regarding internalization of mycobacteria and their role as APC, and extend the knowledge about AEC by assessing the secretion of a broad array of factors in response to several different types of stimuli. We show that AEC respond by producing distinct secretory profiles to the different stimuli, revealing a multifunctional cell type that is likely to play a prominent role in initiating and shaping in situ immune responses. All this provides evidence on a very active role of epithelial cells in the immunological response in the respiratory tract and also provides indirect evidence on the effect of AEC in Mtb infections. Thus, probably in an in vivo situation AEC may modulate responses of other cell populations in the lungs. The principal role of AEC could be to maintain the homeostatic balance of immune response and at the same time, to coordinate the responses to pathogens such as Mtb. A more complete understanding of the role of AEC in promoting cell migration and the subsequent maturation/differentiation of incoming cells remains to be determined.

## Materials and Methods

### Ethics Statement

All mouse experiments were approved and performed in accordance with the guidelines of the Animal Research Ethics Board at Stockholm University (ethical approval ID: N27/10).

### Mice

The studies were performed using 8–12-week-old female C57BL/6 mice purchased from Scanbur AB, Sollentuna, Sweden and housed in pathogen-free conditions. All animals were kept at the Animal Department of the Arrhenius Laboratories, Stockholm University, Sweden. Mice were supervised daily and sentinel mice were used to assess and ensure pathogen free conditions in the facility.

### Bacteria

*M. bovis* BCG (Pasteur strain) obtained from A. Williams, HPA, Salisbury, UK was grown in Middlebrook 7H9 broth (DIFCO, Sparks, MD, USA) supplemented with albumin-dextrose-catalase (ADC), 0.5% glycerol and 0.05% Tween 80 (vol/vol). BCG was collected at a log phase of growth (absorbance 1.0 measured at OD_650_) for a culture period of 10–15 days at 37°C. Aliquots were frozen in PBS with 10% glycerol and kept at −70°C. Three vials picked randomly from the stock were thawed, serially diluted in plating buffer (PBS with 0.05% Tween-80 [vol/vol]) and CFU counted 2–3 weeks after plating on Middlebrook 7H11 agar (Karolinska Hospital, Solna, Sweden) prepared with glycerol, oleic acid-albumin-dextrose-catalase (OADC) and the antibiotics polymyxin B and amphotericin B.

For a rapid quantification of BCG in our cultures, we used GFP-BCG. To construct the GFP-BCG strain, *M. bovis* BCG was transformed with the dual reporter plasmid containing the human codon-optimised and fluorescence-enhanced EGFP and the luxAB genes from *Vibrio harveyi* [21]. This is very convenient since bacteria contents can be quantified immediately by luminescence while the classical evaluation of BCG growth in agar plates takes between 2–3 weeks. Luminescence is expressed as RLU. To determine the RLU, Decanal (Sigma-Aldrich) was used as a specific substrate for the bacterial enzyme luxAB. Decanal was dissolved in 70% ethanol and added to the lysates at a final concentration of 0.01%. The samples were mixed immediately and luminescence was measured after 15 seconds in a Modulus, Turner Bio Systems luminometer.

We performed parallel measurements of bacterial determinations using both luminescence and counts of CFU. Both methods correlated well with bacterial growth (Figure S1) and for convenience only luminescence is shown in this study.

### *In vitro* infection

AEC and PuM primary cells and cell lines were plated at 1×10^5^ per well in 24 well-plates (Costar, NY, USA). Twenty four hours before infection, the medium was replaced with complete RPMI without antibiotics. The MOI of GFP-BCG versus cells was 10∶1 (for RLU and CFU determinations) or 100∶1 (for microscopy studies). Infection time was 4 h. After infection, cells were gently washed three times, and later treated with gentamicin (100 µg/ml) for 1 h at 37°C. This procedure was necessary to assure a complete removal of extra cellular bacteria (data not shown). After treatment, it was equally important to completely remove gentamicin from the cultures by thorough washing to avoid interference with intracellular killing [37].

Infected cells were lysed immediately (0 h) to evaluate bacterial uptake or incubated in complete RPMI or DMEM without antibiotics for 72 h to measure intracellular bacterial growth. Infected cells were lysed with Triton X-100 (Sigma, St. Louise, MO, USA) for 15 min at 37°C to release bacteria from the cells. After that, bacterial growth was measured as described above. Cell viability of infected cells was assessed after 72 h culture using Trypan blue exclusion and the cell viability was over 98%.

For fluorescence microscopy, the cells were cultured on cover glass (VWR International Ltd) and infected as described above. Cells on cover glasses were fixed at 0 h and at 48 h with 4% paraformaldehyde. GFP-BCG inside of the infected cells was visualized in the fluorescence microscope.

### Cell lines

The mouse cell lines MH-S and T7 were purchased from European Collection of Cell Cultures (ECACC, Salisbury, UK). The mouse type II AEC line T7 was maintained at 33°C, 5% CO_2_ in the presence of human IFN-γ 100 U/ml and differentiated as described previously [38]. The MH-S mouse alveolar macrophage cells, SV40 transformed, were grown at 37°C, 5% CO_2_ in complete RPMI medium containing 10% fetal calf serum, 2 mM L-glutamine, 100 U/ml penicillin, 100 µg/ml streptomycin, 2 mM sodium pyruvate and 0.05 mM 2-ME (all from Invitrogen, Paisley, UK). These conditions were used for the rest of cell cultures if not otherwise stated.

### Isolation of AEC and PuM

Primary AEC were prepared from C57BL/6 mice using the Corti's protocol with some modifications [39]. Dispase (Gibco-Invitrogen, Paisley, UK) was instilled into the lung via the trachea; the lungs were removed and incubated in a dispase-containing solution for 45 min at room temperature. The parenchymal tissue was carefully teased apart, and the cell suspension treated with DNAse I (Sigma). The cells were passed through 70 and 40 µm nylon cell strainers (BD Falcon, USA). After treatment with RBC Lysing Buffer (Sigma), the leukocytes were depleted with anti-CD45 microbeads following the manufacturer's protocol using an LS MACS separation column (Miltenyi Biotec, Bergisch Gladbach, Germany). Subsequently, the CD45**^−^** cells were depleted of contaminating endothelial cells using anti-CD146 microbeads (Miltenyi). The remaining CD45^−^CD146^−^ cells were considered to be AEC and were incubated for 48 h and washed to remove non-adherent cells and debris. To obtain PuM, the CD45^+^ cells were incubated for 48 h, and washed to remove non-adherent cells and debris. In average, 98% of the cells were positive for the F4/80 macrophage marker.

### Flow Cytometry

Freshly isolated AEC and PuM were incubated with anti CD16/32 (Mouse BD Fc Block™) from BD-Bioscience Pharmingen, San Diego, CA at a concentration of 1 µg/10^6^cell for 20 min at 4°C. For surface staining, PuM were incubated with anti-mouse F4/80-APC (AbD serotec, Dusseldorf, Germany), AEC were incubated with rat anti-mouse CD45-PE (BD-Bioscience), rat anti-mouse CD31 (PECAM-1)-PE-Cy7 (eBioscience, Hatfield, UK) or their respective isotype controls for 30 min at 4°C. For intracellular staining, cells were fixed with 4% paraformaldehyde for 10 min at room temperature and washed once with PBS. After fixation, cells were permeabilized with saponin buffer (0,2% saponin (Sigma), 0,1% FCS in PBS) for 10 min and washed once with saponin buffer followed by incubation with hamster anti-mouse podoplanin-PE (eBioscience), rat anti-mouse CD74-FITC (BD-Bioscience) or their respective isotype controls for 30 min at 4°C for 30 min at. All samples were analyzed on a Becton Dickinson FACScalibur and data analyzed using CellQuestPro software (Becton Dickinson Immunocytometry Systems). All the analyses were performed with an acquisition of 10000 events.

### Determination of antigen presentation by AEC and PuM

C57BL/6 animals were immunized s.c. at the dorsal neck region three times at two weeks interval with the mycobacterial antigen 19 kDa (10 µg/animal) formulated with 1 µg/animal of cholera toxin (CT) (Quadratech Ltd, Surrey, UK) as adjuvant or left unimmunized. The recombinant mycobacterial antigen (Ag) 19 kDa was obtained from Lionex Diagnostics & Therapeutics GmbH, Braunschweig, Germany. To assess the ability of AEC to act as APC, cells were pulsed with 20 µg/ml of Ag19 kDa for 24 h. PuM were used for comparison. After this, the cell monolayers were washed three times to completely remove free antigen. We named these pulsed cells AEC_19 kDa_ and PuM_19 kDa_, respectively. Spleen cells from unimmunized or immunized animals plated at 5×10^5^ cells/well in 96-well flat bottom plates were cultured together with AEC_19 kDa_ and PuM_19 kDa_ for 72 h at a ratio of 10∶1 (splenocytes: AEC or PuM). As positive controls, splenocytes from unimmunized and immunized animals were also stimulated *in vitro* with 5 µg/ml of Ag19 kDa or with 4 µg/ml of the polyclonal T cell activator concanavalin (Con) A, obtained from Sigma (data not shown). Supernatants were collected and stored at −20°C until tested for IFN-γ content.

### *In vitro* stimulation

AEC and PuM primary cells and cell lines were plated at 5×10^4^ cells per well in 96 well-plates and stimulated for 24 h with various stimuli namely, bacterial LPS (Sigma), Pam3, (Enzo Life Sciences, Lausen, Swizerland), flagellin (*Salmonella.typhimurium*, InvivoGen, San Diego, CA, USA), TNF (R&D systems, Abbington, UK), IFN-γ (Mabtech, Nacka Strand, Sweden), IFN-α (Kindly provided by Alfa Wasserman, Pescara, Italy) and the mycobacterial products heat killed (HK)-BCG, BCG lysate (Lys-BCG). HK-Mtb and HK-SO2 were kindly provided by C. Locht, ISERM, Institut Pasteur de Lille, France. The amounts used were as follows: LPS, 10 µg/ml; Pam3, 1 µg/ml; flagellin, 50 ng/ml; TNF, 10 ng/ml; IFN-γ, 20 ng/ml; IFN-α, 1000 U/ml; HK-BCG, HK-Mtb, HK-SO2, amount corresponding in CFU to 10 times cell numbers; Lys, 10 µg/ml amount also corresponding in CFU to 10 times cell numbers. Culture supernatants were collected and stored at −80°C until tested.

To prepare HK-BCG, 10^7^ CFU/ml of BCG were centrifuged at 8000 g, resuspended in 0.05% Tween-80 in PBS, washed twice and finally autoclaved at 121°C for 15 min. For the preparation of lysate, 10^7^ CFU/ml bacteria were pelleted by spinning at 8000 g, resuspended in 0.05% Tween-80 in PBS, and washed twice. The bacteria were then resuspended in 5 ml of ice cold PBS and sonicated (Sonifier B12; Branson Sonic Power, Danbury, CT, USA) on ice for 14 cycles of 1 min each, as described previously [40]. To remove particulate matter, the sonicated suspension was spun at 12000 g for 30 min at 4°C and the supernatant containing soluble antigens (referred to as Lys-BCG) was collected and stored at −20°C. The protein concentration of the lysate was determined using the Bio-Rad protein assay reagent (Bio-Rad Laboratories, Carlsbad, CA, USA), according to the manufacturer's instructions. Soluble bovine serum albumin fraction V was used as a protein standard.

### Cytokine and chemokine determinations

The profile and content of various cytokines, chemokines and growth factors were measured in cell culture supernatants from untreated and stimulated PuM and AEC by ELISA and a mouse cytokine array panel A (Proteome Profiler™) (R&D Systems) according to the manufacturer's instructions. For ELISA determinations, the commercially available kits for CCL-1/MCP-1 (eBioscience), TNF, matrix metalloproteinase (MMP)-9, CXCL2/MIP-2, CXCL-1/Keratinocyte cell-derived chemokine (KC), GM-CSF, M-CSF, IL-6 CXCL10/Interferon gamma-induced protein 10 kDa (IP-10) and CCL5/RANTES (R&D Systems), IL-12, and IFN-γ (Mabtech) were used to determine the cytokine levels in the culture supernatants according to the manufacturer's recommendations. The enzyme-substrate reaction was developed using p-nitrophenyl phosphate (Sigma) for IL-12, IL-10 and IFN-γ and tetramethylbenzidine substrate (R&D Systems) for the rest of determinations. Depending on the substrate used, the optical density was measured in a multiscan ELISA reader (Anthos Labtech Instruments, Salzburg, Austria) at 405 or 450 nm. The concentrations were calculated from the standard curves established with corresponding purified recombinant mouse cytokines, chemokines and diffusible factors.

For analysis with the Proteome Profiler, spots were developed using Pierce® ECL Western Blotting Substrate (Thermo Scientific, Rockford, IL, USA) and the chemiluminescence measured using a luminescent image analyzer (Fujifilm, LAS-100 plus). Spot size was determined using ImageJ software (NIH, Bethesda, MD, USA).

### Statistical analysis

Data are expressed as means ± SEM or SD. Comparison between the experimental groups was done by unpaired Mann Whitney test. *p* value of <0.05 was considered as the level of significance. One-way ANOVA was used to analyse differences between groups receiving different stimuli, followed by Bonferroni's Multiple Comparison Test. All data were analyzed using the GraphPad InStat version 5.0 (GraphPad Software, San Diego, CA, USA).

## Supporting Information

Figure S1 **Correlation of bacterial growth measurements by luminescence and colony forming units (CFU).** The GFP-BCG bacteria were prepared at different dilutions and quantified using relative luminescence units (RLU) and CFU as described in Materials and Methods. The graph displays both measurements and shows the correlation of both methods.
(TIF)
