# Supplementary figures and images for: The Role of Alveolar Epithelial Cells in Initiating and Shaping Pulmonary Immune Responses: Communication between Innate and Adaptive Immune Systems
Source: PLoS One. 2012 Feb 29;7(2):e32125. doi: 10.1371/journal.pone.0032125 (PMC3290547; doi:10.1371/journal.pone.0032125)

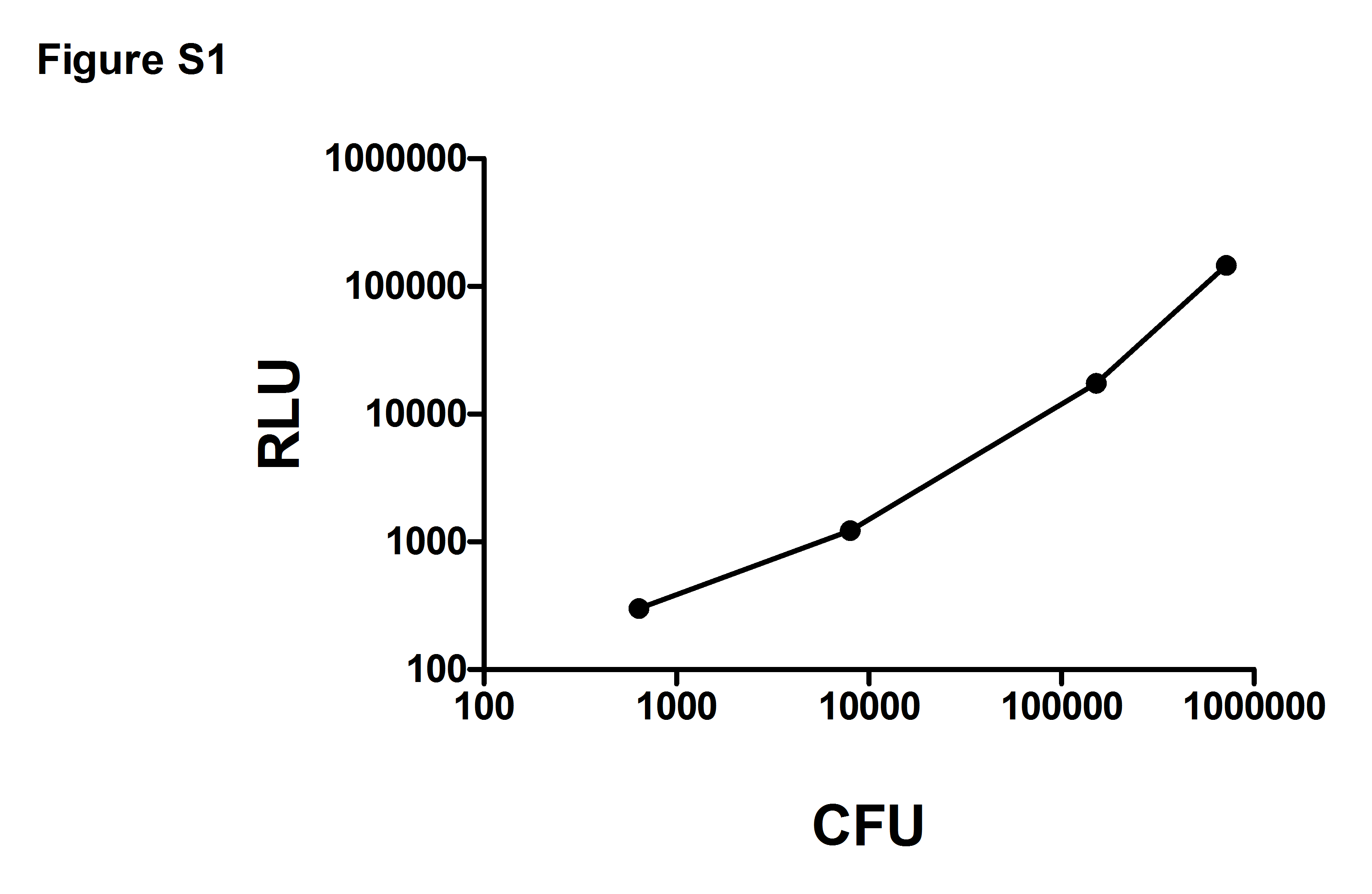

Supplement: Figure S1 — Correlation of bacterial growth measurements by luminescence and colony forming units (CFU). The GFP-BCG bacteria were prepared at different dilutions and quantified using relative luminescence units (RLU) and CFU as described in Materials and Methods. The graph displays both measurements and shows the correlation of both methods. (TIF) [file pone.0032125.s001.tif]
